# Enhanced Figures of Merit for a High-Performing Actuator in Electrostrictive Materials

**DOI:** 10.3390/polym10030263

**Published:** 2018-03-03

**Authors:** Nellie Della Schiava, Kritsadi Thetpraphi, Minh-Quyen Le, Patrick Lermusiaux, Antoine Millon, Jean-Fabien Capsal, Pierre-Jean Cottinet

**Affiliations:** 1 Univ Lyon, INSA-Lyon, LGEF, EA682, F-69621, Villeurbanne, France; nellie.dellaschiava@insa-lyon.fr (N.D.S.); Kritsadi.Thetpraphi@insa-lyon.fr (K.T.); minh-quyen.le@insa-lyon.fr (M.-Q.L.); jean-fabien.capsal@insa-lyon.fr (J.-F.C.); 2 Groupement Hospitalier Edouard Herriot, 69003 Lyon, France; patrick.lermusiaux@insa-lyon.fr (P.L.); antoine.millon@insa-lyon.fr (A.M.); 3 Université Claude Bernard Lyon 1 (Univ Lyon), 8 Avenue Rockefeller Lyon, F-69621 Villeurbanne, France

**Keywords:** electrostrictive unimorph cantilever, deflection, blocking force measurement, actuators, material optimization, figure of merit

## Abstract

The overall performance of an electrostrictive polymer is rated by characteristic numbers, such as its transverse strain, blocking force, and energy density, which are clearly limited by several parameters. Besides the geometrical impact, intrinsic material parameters, such as the permittivity coefficient as well as the Young’s modulus and the breakdown electric field, have strong influences on the actuation properties of an electroactive polymer and thus on the device’s overall behavior. As a result, an analysis of the figures of merit (FOMs) involving all relevant material parameters for the transverse strain, the blocking force, and the energy density was carried out, making it possible to determine the choice of polymer matrix in order to achieve a high actuator performance. Another purpose of this work was to demonstrate the possibility of accurately measuring the free deflection without the application of an external force and inversely measuring the blocking force under quasi-static displacement. The experimental results show good electrostrictive characteristics of the plasticized terpolymer under relatively low electric fields.

## 1. Introduction

Electrostriction is a common phenomenon encountered in dielectric materials placed under the influence of an external electrical field; it consists of the generation of stress and strain [1,2,3]. Normally, any dielectric material is electrostrictive by nature, but strain/stress effects are more pronounced in some materials [4]. Among them, polymers are an interesting class due to their flexibility, light weight, and high permittivity, leading to the generation of considerable strain, which can be very promising in various actuator applications, like micro-pumps [5], haptic devices [6], smart guidewires for medical instruments [7], and so on. Nevertheless, the electrical fields required to reach significant deformations are relatively high (hundreds of V/μm) [8]. For this reason, researchers are currently focusing on the synthesis of new polymers with enhanced electrostrictive properties, making it possible to drastically reduce the applied electric fields [9,10]. Other alternative strategies leading to drastically enhanced electromechanical performances of the electroactive materials are also being investigated, such as the use of a model of swollen elastomers based on interpenetrating networks which contains certain instabilities [11], or by conducting microstructure compositions by means of numerical simulation with the aid of both idealized and periodic models [12].

As recently reported [13], one of the simplest practical techniques is to incorporate a plasticizer into the existing polymer matrix. Excellent results in terms of the transverse strain as well as the energy density under a very low electric field have been achieved. An essential step of such an approach is to be able to compare the performance of different materials in order to find the best chemical composition. Such a comparison should be based on a standard method that can be widely applied to any kind of electroactive polymer, leading to a simple way of choosing an adequate actuator for a specific application. This issue should be particularly considered in the material optimization phase, but unfortunately, there is no mention of it in the literature. For instance, the strain versus electric field for a polymer is generally used to evaluate the actuator’s quality. However, this criterion is far from sufficient as the polymer film can display extremely large deformations but may be very soft, resulting in undesirable effects like buckling or poor mechanical coupling that deteriorate the device’s performance. Another approach found in the literature revolves around the actuation behavior of an electroactive polymer, principally depending only on the electrostrictive coefficient [14,15]. For the same reason, this criterion was not suitable for all actuator applications. Accordingly, in addition to analyses on the strain response and electrostrictive coefficient, other material parameters, like the Young’s modulus, the electric field limit, etc., should be discussed, so as to find a good trade-off between force and strain characteristics in order to achieve appropriate actuator behaviors.

Based on the above arguments, this paper reports on a simple method for assessing the electroactive material properties based on various characteristics, such as the force, the strain, and/or the energy density. In other words, the proposed method aims to integrate different materials in a particular actuator and to measure its effectiveness by means of defined standard criteria for the figures of merit (FOMs). The study also explains that the characteristics are strongly affected by intrinsic material parameters, including the permittivity and the Young’s modulus as well as the electrical breakdown. Another objective of this paper was to provide an efficient technique for accurately measuring the blocking force under a quasi-static regime. The precision of the force measurement clearly depends on the type of load cell as well as on the manner in which it is installed on the actuator. Due to these reasons, empirical force measurements are quite challenging and rarely reported in the literature, even though they are considered to be some of the most important factors reflecting the actuation performance of an electroactive polymer. Indeed, a performance actuator is not only qualified by how much displacement it can achieve but also how much load it can exert. As a result, this paper proposes a general method for evaluating the electroactive characteristics based on the FOMs of force, strain, and energy density, making it much easier to compare different kinds of materials. 

This paper is organized as follows: Section 2 aims to provide a method based on figures of merit that embraces the material parameters that affect their behavior. Other issues related to the electrical breakdown as well as a selection of polymer matrixes are also discussed. Section 3 is dedicated to the actuator’s preparation and the test bench setup, followed by experimental results described in Section 4. Finally, the conclusion and future works are presented in Section 5.

## 2. Method for Achieving High Material Performances

### 2.1. Figures of Merit Based on an Electrostrictive Material Model

The well-known model for an electrostrictive material can be written as follows [16]:

(1) $$\left\{ \begin{array}{l} S_{31}^{}=M_{31}^{}E_{}^{2}+s_{}^{E}T_{31}^{}\\ D_{31}^{}=\varepsilon E+2M_{31}^{}ET_{31}^{} \end{array}\right.$$

where *S* is the strain, *T* is the stress, *D* is the electric displacement, *E* is the electric field, *M* is the electrostrictive coefficient, *s* is the compliance, and *ε* is the permittivity of the polymer.

**Figure 1:**
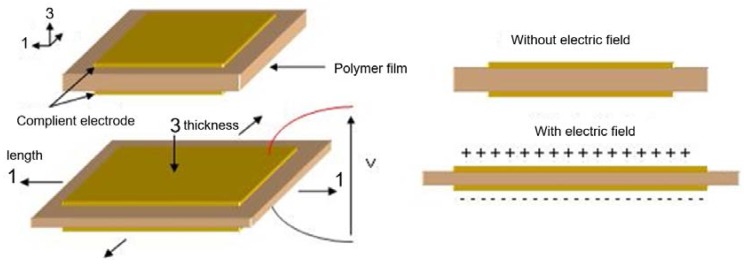
Principle schematic of the electrostrictive actuation.

It is noteworthy that *S*_31_ denotes the transverse strain corresponding to the ratio of change in the length of the sample (direction of axis 1) under an applied electric field along the thickness direction (axis 3). A similar explanation can be made for other parameters, like *T*_31_ and *D*_31_. Figure 1 illustrates the direction of the applied electric field as well as the deformation.

Without stress (i.e., for *T*_31_ = 0), the electrostrictive transverse strain under an electric field can be simplified as [17]:

(2) $$S_{31}^{}=M_{31}^{}E_{}^{2}$$

where the electrostrictive coefficient, *M*_31_, depends on the permittivity and the Young’s modulus of the material, as expressed in Equation (3):

(3) $$M_{31}^{}\propto \frac{\varepsilon_{0}^{}\varepsilon_{r}^{}}{Y}$$

Here, *ε_r_* is the relative permittivity of the polymer and *ε*_0_ is the vacuum permittivity. 

From Equations (2) and (3), it is noteworthy that the resulting strain under a constant electric field can be enhanced by either increasing the permittivity or decreasing the Young’s modulus. Therefore, the FOM of the strain is defined by

(4) $${\mathrm{FOM}}_{\mathrm{strain}}=\frac{\varepsilon_{0}^{}\varepsilon_{r}^{}}{Y}$$

Substituting *S*_31_ = 0 in Equation (1) yields:

(5) $$T_{31}^{}=YM_{31}^{}E_{}^{2}$$

Combining Equations (3) and (5) makes it possible to induce the FOM of the blocking force for a given electric field

(6) $${\mathrm{FOM}}_{\mathrm{force}}=\varepsilon_{0}^{}\varepsilon_{r}^{}$$

Again, the above equation demonstrates a further advantage of increasing the permittivity in order to achieve a better actuation performance. 

Besides the strain and the blocking force, another critical parameter also affecting the electromechanical coupling of the polymer is the mechanical energy density (*W_m_*) which is given by the following expression [13]:

(7) $$W_{m}^{}=\frac{1}{2}YS_{31}^{2}$$

Substituting Equation (2) into (7) yields:

(8) $$W_{m}=\frac{1}{2}\frac{{\left(\varepsilon_{0}^{}\varepsilon_{r}^{}\right)}_{}^{2}}{Y}E_{}^{4}$$

Finally, the FOM of the mechanical energy density can be defined as:

(9) $${\mathrm{FOM}}_{\mathrm{energy}}=\frac{{\left(\varepsilon_{0}^{}\varepsilon_{r}^{}\right)}_{}^{2}}{Y}$$

**Figure 2:**
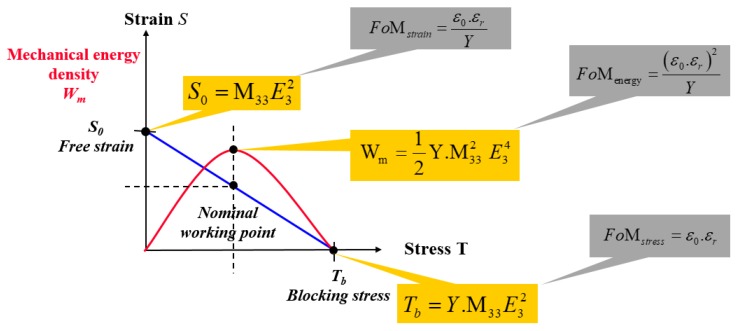
Pertinent parameters and figures of merit for an electroactive polymer.

In actuator applications based on electroactive polymers, the strain response, the blocking force, and the mechanical energy density under a low electric field can be simultaneously enhanced by increasing the dielectric permittivity of the polymer. For a better electromechanical coupling, especially in low-frequency actuator applications, a decrease in the Young’s modulus should be involved but it must be limited in order not to drastically change the elasticity or the compliance of the material. Figure 2 summarizes the FOM of an electrostrictive actuator based on three critical parameters comprising the strain response, the blocking force, and the mechanical energy density.

### 2.2. Electrical Breakdown

An ideal electrostrictive material does not only have a high figure of merit, but also an electrical breakdown that occurs when the dielectric strength of the material is exceeded. In practical situations, each material has its ideal electric field for operation that directly affects the limits of the strain range.

**Figure 3:**
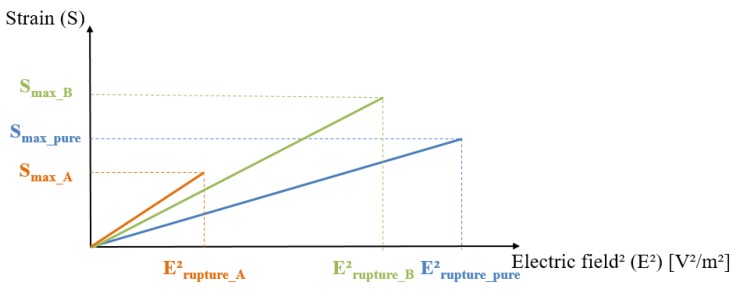
Strain versus electric field squares for different electrostrictive composites.

To better clarify these issues, we here introduce three different kinds of composites, i.e., a pure polymer and two modified polymers (A and B) possessing higher electrostriction coefficients than the pure polymer but with different electric field breakdowns. Figure 3 illustrates the linear relationship between the strain and the electric field square for these three composites, where the slope of each curve represents the electrostriction coefficient, *M*_31_. As observed, the *M*_31_ value of sample A is higher than for the other samples, leading to a better strain response for a given electric field. Nonetheless, its electrical breakdown is relatively low, drastically reducing the strain range and, as a result, limiting the electromechanical conversion. This kind of material is suitable for applications where a low input voltage is mandatory. The pure material, on the other hand, exhibited a large range of electrical breakdown but had a quite poor electrostrictive property and required an enormously high input voltage to attain satisfactory strain. 

Sample B seemed to be a good compromise between the electrostrictive behavior and the electrical breakdown limit. However, we cannot conclude that this material is the most appropriate choice in any practical application, nor that material A, with its higher electrostrictive coefficient, would be. Again, it should be kept in mind that the choice of each material is strongly affected by the intended use. For a better improvement in the electromechanical properties, it is important to ensure a fair balance between the various parameters, including the strain, the blocking force, the electric field range, etc. The weight given to each criterion effectively depends on the application area as the final purpose is not only to achieve the greatest electrostrictive behavior, but also to have a reasonable electric field range.

Finally, this paper proposed to compare the characteristics of different electrostrictive materials to those of a standard material (e.g., the pure polymer). All criteria should be based on the FOMs of the strain, the blocking force, and the energy density as well as the allowable maximal voltage imposed by the final application. 

### 2.3. Selection of a Polymer Matrix

In this section, we justify how to choose an electroactive material suitable for an actuation application based on several criteria presented previously. As reported in [18], the main drawback of most electrostrictive polymers and dielectric elastomer actuators is their high electric field requirement, i.e., in the 1 kV range. To overcome this limitation, a recent innovative solution reported by Capsal et al. [13] involved enhancing the electromechanical performance of a P(VDF-TrFE-CTFE) fluorinated terpolymer by doping it with a plasticizer (e.g., DEHP or DEIP), giving rise to a decreased Young’s modulus and an increased dielectric permittivity of the material [19]. This simple chemical modification made it possible to achieve excellent electrostrictive strain with an electric field of around 5.5 times lower than that required with a conventional terpolymer. 

Table 1 depicts the characteristics of different electroactive polymers, including the modified terpolymer and conventional polymers. It is clear that the modified terpolymer presented excellent results in terms of force, strain and mechanical energy density, with its figures of merit largely superior to those of the other polymers. On the other hand, the breakdown field of the modified sample was limited at around a hundred V/μm, but this is generally sufficient for most actuation applications where the input voltage must be restricted. Finally, using a simple method of adding a reasonable quantity of plasticizer into the terpolymer matrix, the novel material invented by [13] allowed a five-fold increase in dielectric permittivity and a three-fold decrease in the Young’s modulus, making it one of the best candidates for actuator applications with an exceptional electromechanical response. 

**Figure 4:**
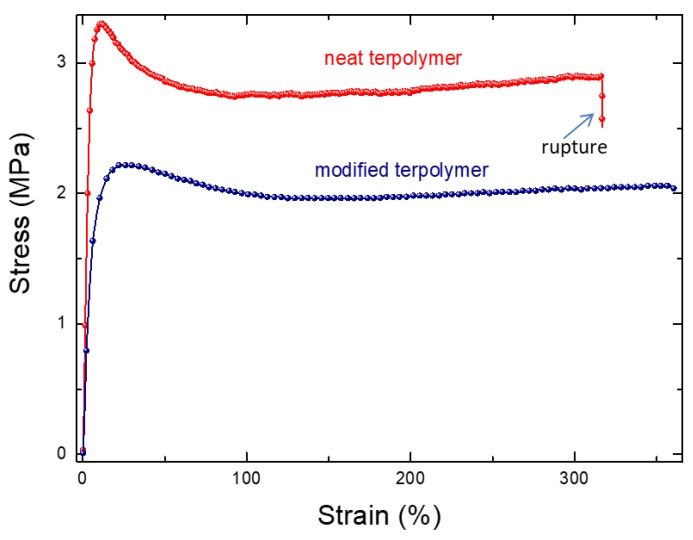
Longitudinal strain versus stress for different electrostrictive composites.

**Figure 5:**
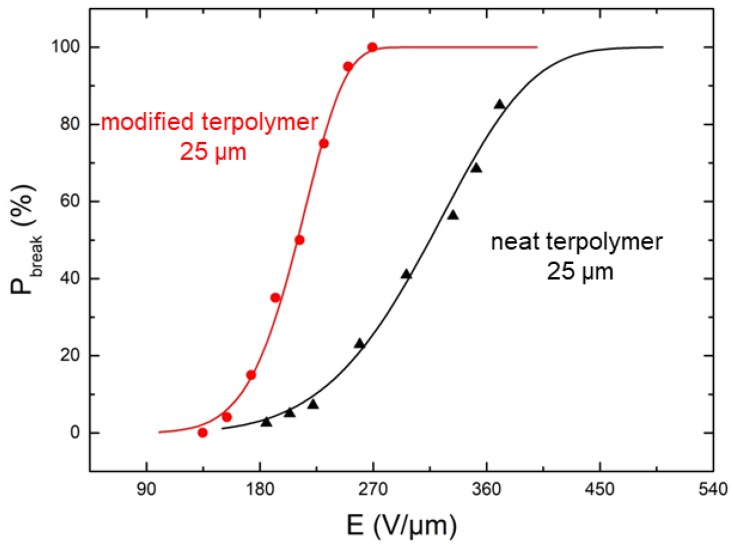
Breakdown probability versus electric field for different electrostrictive composites.

**Figure 6:**
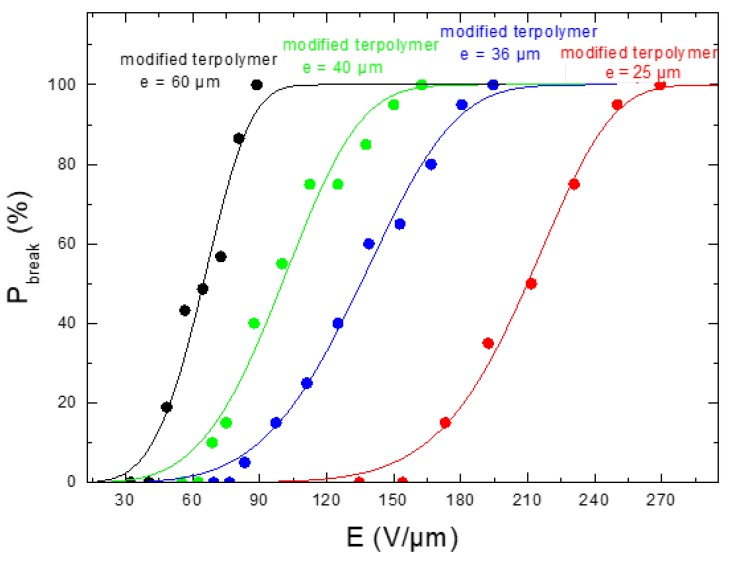
Breakdown probability versus electric field of modified terpolymers with different thicknesses.

To better assess the performance of the plasticized polymer, the mechanical properties characterizing the material rupture based on the stress/strain behavior, along with the electrical breakdown as a function of the input electric field, were investigated. It is shown in Figure 4 that the modified sample gives rise to a higher critical strain level, which is in agreement with the fact that the Young’s modulus of the neat terpolymer is superior to the modified one. The results in Figure 5, on the other hand, showed the limitations of the modified polymer, in which the probability of material breakdown is more significant, compared to the pure polymer under the same electric field. Figure 6 highlighted the dependence of the material’s thickness on the breakdown probability. As expected, the modified sample with the smallest thickness of 25 μm led to the highest allowable electrical field, justifying the choice of this configuration in the next study. Accordingly, the plasticized terpolymer seems to be very promising for flexible actuator devices where a low input voltage is needed. Finally, the electrical and mechanical properties can be imperative factors that may limit or promote future applications of material design.

In the following sections, the modified terpolymer was chosen to assess the electrostrictive performance based on the criteria described above. Comparison of the FOMs of force, strain, energy density as well as the electric field range of this polymer and other former materials were also investigated. 

## 3. Fabrication Process and Characterization Test Bench

### 3.1. Sample Preparation

As the manufacturing of electroactive polymer devices is quite simple, there are many possibilities for sample’s fabrication available. The most commonly used configuration consists of a top metallic layer, an adhesive layer, and finally, a polymer layer. Electroactive polymer devices also vary in shape and thickness, which may lead to different behaviors that are strongly affected by the boundary conditions [4,23]. Also, the type of mounting conditions highly influences the resonant frequency and the electromechanical coupling.

This paper describes the use of a new all-organic composite, proposed by Capsal et al. [19]. It consists of a P(VDF-TrFE-CTFE) 56.2%/36.3%/7.5% terpolymer powder, provided by Piezotech S.A.S (Arkema group, Lyon, France). Films were made by solution casting. Firstly, the terpolymer was dissolved in a 14%wt. Methyl Ethyl Ketone (MEK, Sigma–Aldrich, Paris, France) solvent. The solution was then cooled at room temperature for 2 h. Secondly, (2-ethylhexyl) phthalate (DEHP, Sigma–Aldrich) was added into the polymer matrix and stirred for 1 h. The mixture was then cast onto a glass substrate using a Doctor blade (Elcometer, Manchester, UK), and the solvent was left to evaporate. The films were subsequently placed in an oven at 60 °C for 12 h to ensure that the solvent was completely removed. In order to enhance the crystallinity of the samples, annealing at 103 °C occurred for one hour. Lastly, a Cressington Sputter Coater (208 HR, Orlando, FL, USA) deposited 25 nm-thickness gold electrodes on each side of the plasticized terpolymer film with 25 μm of thickness.

To prepare the electroactive composite actuator, a sheet of the P(VDF-TrFE-CTFE) composite was cut into two strips of 40 mm × 10 mm. A PolyEthylene Terephthalate substrate (PET) with the same dimensions as the polymer film was prepared and an adhesive layer was then applied between the terpolymer and the Mylar substrate. The PET was chosen due to its interesting characteristics, like flexibility, simple implementation, commercially available, and low cost. In addition, it is widely used as a substrate in various printed electronics. These features are very interesting for applications of printing multilayer design which aim to reduce the power supply voltage.

**Figure 7:**
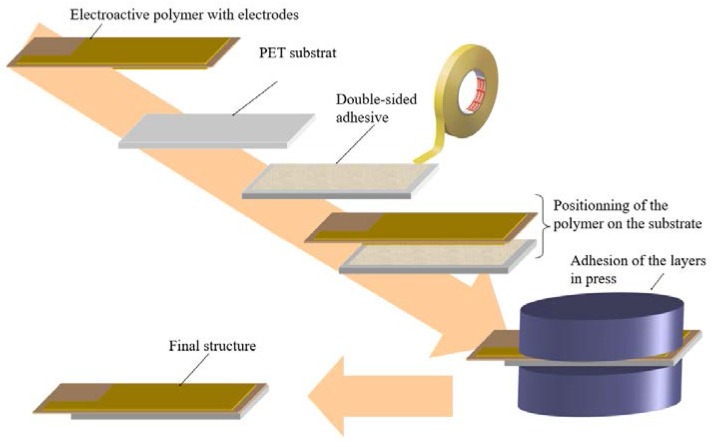
Fabrication process of a P(VDF-TrFR-CTFE) composite actuator.

Finally, the assembly was pressed to increase the interfacial bonding between the active layer and the substrate. Figure 7 illustrates the fabrication procedure of the bender actuators. This actuator fabrication process was relatively simple compared to that of carbon nanotube Buckypaper actuators [24].

### 3.2. Experimental Setup of the Actuation Test

This section deals with the influence of a measurement apparatus on the results. For complete characterization of the actuator, the displacement as well as the generated force must be measured.

By definition, “free stroke” is the actuator’s displacement under zero external load [25]. Measurements of the “free stroke” are considered to be the most elementary of the actuation characteristics. However, depending on the boundary conditions, results may vary greatly. Cantilever measurements under no load present no difficulty, but for unimorph actuators, several factors need to be taken into account. Holding the active parts of the actuator will lead to slightly different results than if the inactive part is held, and this also depends on where and how the actuator is held. For instance, it is usually specified that the unimorph should be held at a particular distance from the edge of the actuator, as the displacement of an electroactive actuator depends upon the applied voltage, frequency, and force.

Displacement measurements are quite simple, as the problem is simply to accurately measure the motion of a specified point on the actuator. Transducer accuracy can vary from quite coarse (e.g., mechanical dial gauges with 0.025 mm precision) to extremely fine (e.g., laser interferometers with high resolution in the micrometer range) and the choice is largely dependent on experimental needs and cost. The instrument must be mounted to limit vibration and minimize interference with actuator motion and environment, but these mechanical limitations can be readily overcome. In our case, the system was mounted on top of the Iso-Plate Passive Isolation System to dampen vibrations in the room. The tip displacement was measured using the non-contact laser sensor (Microtrak II; MTI Instrument, Inc., Berling, Germany). The contact-type instrument was not employed in our device in order to limit inertial effects that may distort the measurements. Furthermore, it was difficult to estimate how the developed forces changed with the presence of an extra mass, particularly when its amplitude depended on the contact type and was evaluated as a function of time. The situation became even more complicated when the frequency was varied.

In contrast to the displacement measure, the cantilevered blocked force measure is much more difficult. Indeed, the accuracy of the force measurement largely depends on the range and resolution of the load cell as well as its integration on the actuator. For this reason, when selecting an actuator, the manner in which the force is measured becomes an issue. The results can be very dissimilar between load cell configurations. Consequently, the conditions surrounding the force measurement and its experimental setup should be well defined. For instance, a definition of cantilevered blocked force is the force observed on a gauge when holding the tip of the actuator during energizing [26]. 

It is noteworthy that integrating the load cell may produce the inertial effect on the actuator. In our case, this phenomenon can be discarded since the system operated under relatively low frequencies. In fact, the operating frequency range was selected in such a way that the inertial force was negligible compared to the actuator’s force, allowing simplification of the interpretation and the performance analysis. Another difficulty regarding force measurement devices stems from the fact that it is not possible to accurately operate without restricting the motion of the actuator. Indeed, a load cell can only move a very short distance over its full measuring range, e.g., 25.4 μm to 100 μm for the FC-22 load cell. Hence, the device must be able to measure the actuator’s force over the full range of displacement without biasing the result, with the force needed to move the device itself. In our case, a Transducer Techniques GSO-10 10 g load cell with a TMO-1 signal conditioner/amplifier was employed since this sensor has a high sensitivity and a large frequency bandwidth.

**Figure 8:**
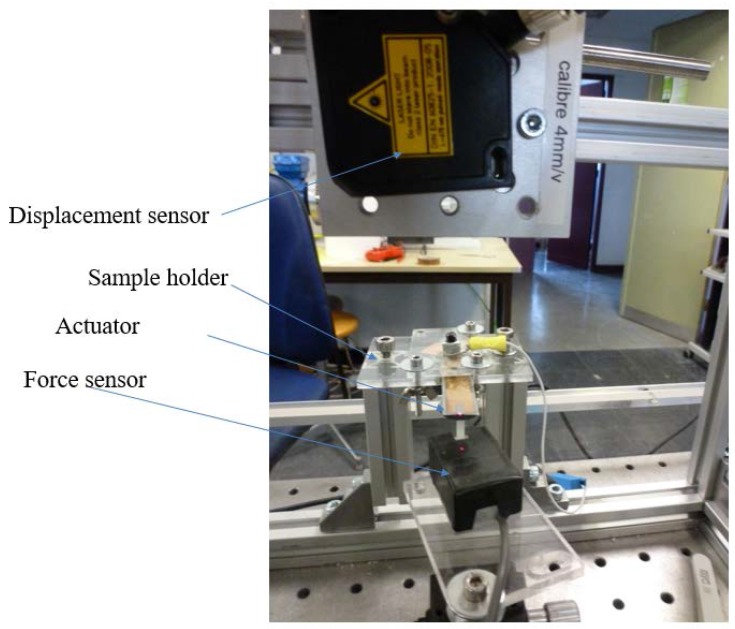
Photo of the test bench.

Finally, in the experimental design for force–displacement measurements of an electroactive polymer actuator, the requirement for adequate equipment was not a trivial problem. Figure 8 shows the essential components of the test bench, including the developed actuator, the load cells allowing the measurement of the blocking force for extremely low displacements of the sample, and the displacement transducers fastened to the upper end of the actuator which made it possible to measure the motion of the bender. To improve the precision of the force measurement, the load cell was mounted on the carriage of a screw-driven slide, making it possible to accurately determine the relative positions of the load cells and the polymer based on the slide’s scale and the graduated dial. The input voltage was generated from the NI 9263 output analog card and was then amplified by a high voltage amplifier (Treck 609D-6 Lockport, NY, USA). The signal was applied to the actuator through the set of compliant electrodes mounted on the fixture base. All the data were monitored with LabVIEW software, Paris, France.

## 4. Results and Discussions

This section describes a set of experiments conducted on actuators made from the neat and DEHP-plasticized terpolymer P(VDF-TrFE-CTFE). In order to assess the performance of these electrostrictive polymers, the electromechanical response was measured as a function of different stimuli under very low frequency. As mentioned above, working at a low frequency made it possible to enhance the accuracy of the force and strain measurements. Another purpose was the extremely high permittivity of the modified terpolymer under such a low bandwidth. For more details about the mechanical and dielectric properties of the proposed material as a function of frequency, the reader can refer to [13]. 

It is noteworthy that, in our experiment, the input electric field was limited to around 40–50 V/μm which is small enough with respect to the breakdown strength of the terpolymer (see Table 1). As reported in [19], a low electric field also enables a fit with the classical Maxwell electrostrictive model and to any non-linearity due to the saturation effect being avoided. The following subsections demonstrate that under such moderate electric fields, high electrostrictive responses in terms of force and strain are still achieved, confirming the excellent actuation characteristics of the proposed materials.

### 4.1. Free Displacement

**Figure 9:**
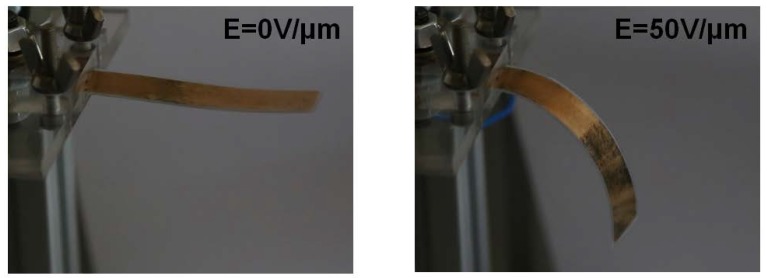
Photo of the unimorph under different input electric field values.

In order to highlight the actuation performances of the all-organic composites, several unimorph architectures were investigated. Figure 9 describes the field-induced actuation of a unimorph comprising an active polymer P(VDF-TrFE-CTFE) + 10 wt % DEHP and an inactive PET substrate. To prevent a discharge effect during operation under high voltage, the surface approximatively 1.5 mm from the edge of the active layer was not covered with an electrode. Figure 9 shows the actuation behavior of the developed unimorph under electric fields of 0 V/μm and 50 V/μm. A high deflection response of the modified terpolymer was achieved under a relatively low electric field.

Based on [27], the tip displacement (*δ*) of a unimorph depends on the device’s geometry as well as the material’s properties. Under quasi-static conditions, the transverse strain (*S*_31_) of a unimorph can be inferred from the deflection measurements, according to the following expression:

(10) $$\delta_{0}=\frac{3L^{2}}{2e}\frac{2AB{\left(1+B\right)}^{2}}{A^{2}B^{4}+2AB(2+3B+2B^{2})+1}S_{31}$$

where *δ*_0_ is the cantilevered tip deflection; *e* and *L* are, respectively, the sample thickness and the length, *Y_substrate_*, *Y_poly_*, *e_substrate_* and *e_poly_* depict the Young’s modulus of the substrate, the Young’s modulus of the polymer, the substrate thickness, and the polymer thickness, respectively, *A = Y_substrate_/Y_poly_* and *B = e_substrate_/e_poly_*.

**Figure 10:**
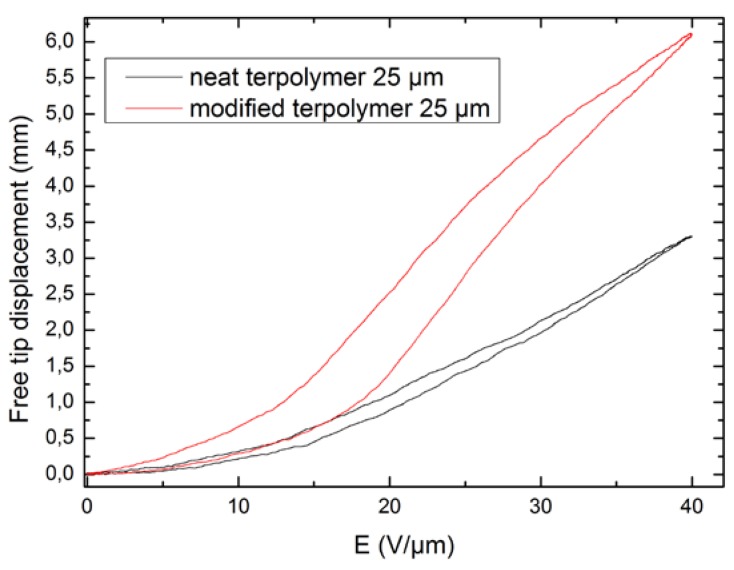
Free displacement versus electric field at 0.1 Hz for two terpolymer compositions.

Figure 10 illustrates the empirical quasi-static displacement versus the electric field at 0.1 Hz for the pure and modified terpolymers. A quadratic dependence of the induced deflection under low electric fields (less than 50 V/μm) was observed, in contrast to the saturation behavior of the deflection at higher input electric fields. As expected, a gap between the ascending and descending branches occurred, representing the hysteresis characteristic of the composite. Similar to other electroactive materials, like magnetic substrates, magnetostrictive materials, piezoceramics, shape memory alloys, etc., when the electrostrictive actuator partially inherits the piezoelectric property, it exhibits the disadvantages of hysteresis and creep (or drift) behaviors [28]. Such comportments may deteriorate the system’s performance and lead to oscillation vibration and even instability, especially where very precise positioning is required, for instance in the case of atomic force microscopes or micro-manipulators. On the other hand, compared with shape memory alloys, the hysteresis of our material was much lower under similar electric field cycling [29,30].

The effect of the DEHP plasticizer on the electrostrictive response of the terpolymer at low a frequency is clearly confirmed by Figure 10, with a 2.5-fold enhancement between the modified and neat terpolymers. It has been previously reported that in the case of dielectric polymers, the electrostrictive strain under an electric field can be mainly attributed to Maxwell forces, induced by dipolar orientation within the material [4]. Also, experimental measurements demonstrated an exceptional increase in dielectric permittivity as well as a somewhat decreased Young’s modulus of the plasticized composite, leading to a large enhancement of the electrostrictive coefficient and the figures of merit.

### 4.2. Blocking Force

**Figure 11:**
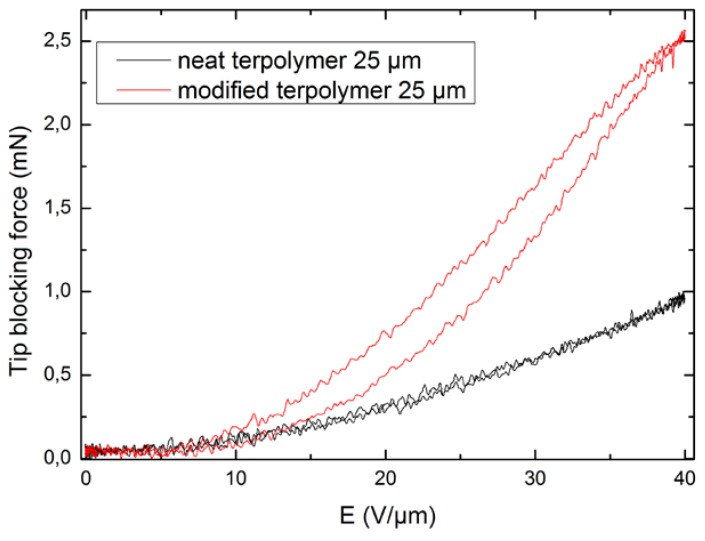
Blocking force versus electric field for two terpolymer compositions.

Figure 11 depicts the evaluation of the measured blocking force as a function of the applied electric field for two compositions of terpolymer. A quadratic dependence of the blocking force in terms of the electric field was achieved, confirming good agreement between experiment and theory, as shown in Equation (5). A comparison between the former and the modified samples demonstrates a great enhancement of the blocking force, with a 2.5-fold increase under 40 V/μm. Such a large improvement can be explained by the fact that the modified material exhibited a higher dielectric permittivity, leading to higher FOM of the blocking force, based on Equation (6).

In conclusion, in order to optimize the force and strain generated by an electrostrictive material, its dielectric permittivity should be increased. This observation correlated with the working principles of the electrostrictive materials since the Maxwell electrostatic stress (*T_E_*), according to Equation (5), induces strain in the material. As a result, an enhancement in force leads to a better strain response and vice versa. These two parameters play a key role in the performance of polymer actuation. 

### 4.3. Force versus Deflection

With electroactive benders, the ability of the actuator to generate force decreases linearly with deflection [31,32]. The experimental force versus the deflection tip were derived as follows: The developed actuator was mounted in a rigid clamp equipped with an electrical contact. The sensing element for the load cell was positioned 1 mm to 2 mm from the end of the actuator. Also, it was placed at a desired distance from the bender’s reference position so that the sample could freely deflect before coming in contact with the load cell. Following excitement from a step electric field, the actuator delivered a force signal whose peak value was recorded by the LabVIEW software. The deflection at which the actuator was unable to exert a measurable force on the load cell was considered the free deflection. Experiments were conducted under 0.1 Hz frequency and four levels of electric field, comprising 10 V/μm, 20 V/μm, 30 V/μm, and 40 V/μm. 

**Figure 12:**
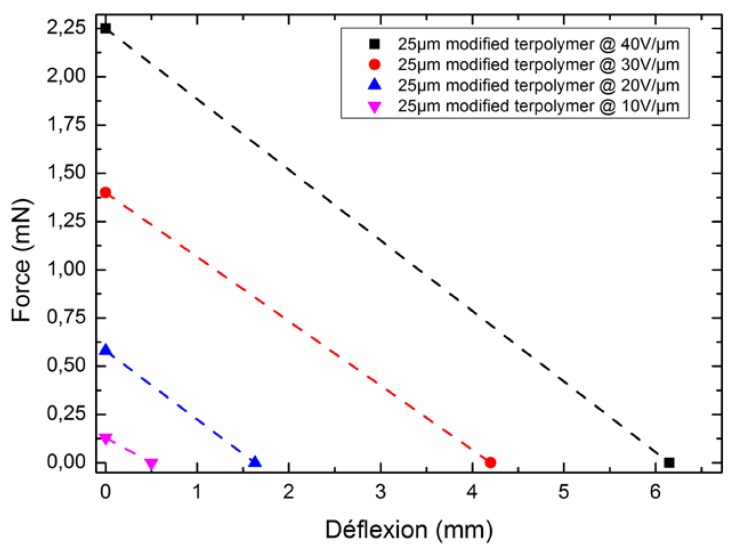
Actuator force versus displacement at 0.1 Hz under different electric fields.

Figure 12 displays the experimental force as a function of the deflection for different input electric fields. As expected, a linear relationship was achieved, representing typical characteristics of the bender actuator. The force at zero deflection is defined as the blocking force, corresponding to the maximum force that the actuator can exert upon an electric field. At the opposite end of the curve, the deflection at zero force is called the free deflection (*δ*_0_). It is then possible to express the deflection (*δ*) function of the curve as follows:

*δ* = *δ*_0_ − *F*/*K* (11)

where *F* is the applied force and *K* is the stiffness coefficient of the actuator.

Based on the force–deflection curve, it is possible to predict the actuator’s displacement as a function of the externally applied load, which is a relevant parameter for designing electroactive material applications. 

### 4.4. Mechanical Energy

The mechanical energy generated by an actuator is also a key parameter reflecting the performance of the electrostrictive polymers. Depending on the position of an object subjected to a conservative force, the mechanical energy is usually defined as the actuator’s ability to carry out work. If *F* represents the conservative force and *x* represents the position, the mechanical energy of the force between the two positions (*d*_1_ and *d*_2_) is defined as

(12) $$E_{mechanic}=\overset{d_{1}}{\underset{d_{2}}{\int }}Fdx=F.\delta$$

**Figure 13:**
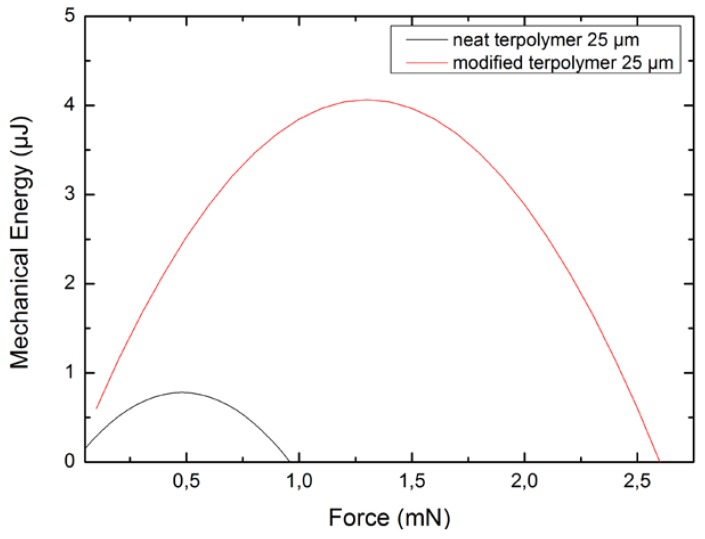
Mechanical energy density function of deflection for two compositions of terpolymer for an electric field of 40 V/μm.

Figure 13 illustrates the mechanical energy generated by two kinds of terpolymer benders as a function of the applied force under a 40 V/μm electric field. The result reveals an optimum point where the mechanical energy passes through a maximum, corresponding to half of the maximum force. It is noteworthy that the mechanical energy of the all-organic plasticized polymer is six-fold that of the neat polymer. These last remarks demonstrate the advantage of using a plasticized composite, since the applied input electric field can be largely reduced to achieve similar electrostrictive properties.

The mechanical energy density can be inferred from the relationship in Equation (7) or from the calculation of the ratio between the energy *E_mechanic_* and the volume. As the volume of the sample is equal to 10^−8^ m^3^, the optimal energy density of the pure terpolymer and its plasticized counterpart respectively reach 70 J/m^3^ and 400 J/m^3^ under a relatively low electric field of 40 V/μm. Again, this result demonstrates that the fact that incorporating a plasticizer into the polymer matrix enables a drastic improvement of the actuation properties as well as a reduction in the input voltage, which is currently one of the major drawbacks of electrostrictive materials.

## 5. Conclusions

This paper has proposed new criteria for assessing the electroactive material performance based on the figures of merit of the force, the strain, and the energy density. These characteristic numbers are greatly affected not only by the intrinsic parameters like the permittivity and the Young’s modulus of material, but also by the breakdown electric field. The proposed approach is an efficient way to select a polymer matrix with large actuation properties and makes it possible to simplify the comparison between different kinds of electroactive composites. Moreover, this work provides an efficient method for achieving highly accurate force measurements which can be quite challenging, especially under quasi-static conditions. The experimental results revealed a large enhancement of electromechanical activities in terms of free deflection and generated force under a relatively low electric field by incorporating a plasticizer into the terpolymer matrix. This phenomenon confirms the high potential of the developed material for real-world actuator applications, especially in multifunctional flexible electroactive devices. Future works will focus on the mechanical behavior of actuators, including displacement and force, over a wide frequency range. Further characterization of the material in various dynamic regimes should also be investigated.

## Figures and Tables

**Table 1:** Characteristics of different electroactive polymers.

Type	PDMS [20]	PU [21]	Nylon [22]	Neat Terpol [13]	Modif. Terpol [13]
ε*_r_* @ 1 Hz	2.5	4.3	5	35	150
Y (MPa)	2	20	2000	100	30
FOM_energy_ (F²/N)	2.44 × 10^−28^	0.72 × 10^−28^	0.01 × 10^−28^	9.58 × 10^−28^	586 × 10^−28^
FOM_force_ (F/m)	2.21 × 10^−11^	3.80 × 10^−11^	4.42 × 10^−11^	30.9 × 10^−11^	133 × 10^−11^
FOM_strain_ (Fm/N)	1.11 × 10^−17^	0.19 × 10^−17^	0.22 × 10^−19^	0.31 × 10^−17^	4.42 × 10^−17^
Breakdown field (V/μm)	200	50	30	150	140

PDMS: Polydimethylsiloxane; PU: Polyrethane; FOM: Figure of Merit.
